# Methionine-mediated gene expression and characterization of the CmhR regulon in *Streptococcus pneumoniae*

**DOI:** 10.1099/mgen.0.000091

**Published:** 2016-10-21

**Authors:** Muhammad Afzal, Sulman Shafeeq, Oscar P. Kuipers

**Affiliations:** ^1^​ Department of Molecular Genetics, Groningen Biomolecular Sciences and Biotechnology Institute, University of Groningen, Nijenborgh 7, 9747 AG, Groningen, The Netherlands; ^2^​ Department of Bioinformatics and Biotechnology, G C University, Faisalabad, Pakistan; ^3^​ Department of Microbiology, Tumor and Cell Biology, , Karolinska Institutet, Nobels väg 16, Stockholm, SE-171 77, Sweden

**Keywords:** Methionine, CmhR, *Pneumococcus*, MetE, MetQ

## Abstract

This study investigated the transcriptomic response of *Streptococcus pneumoniae* D39 to methionine. Transcriptome comparison of the *S. pneumoniae* D39 wild-type grown in chemically defined medium with 0–10 mM methionine revealed the elevated expression of various genes/operons involved in methionine synthesis and transport (*fhs*, *folD*, *gshT*, *metA*, *metB-csd*, *metEF*, *metQ*, *tcyB*, *spd-0150*, *spd-0431* and *spd-0618*). Furthermore, *β*-galactosidase assays and quantitative RT-PCR studies demonstrated that the transcriptional regulator, CmhR (SPD-0588), acts as a transcriptional activator of the *fhs*, *folD*, *metB-csd*, *metEF*, *metQ* and *spd-0431* genes. A putative regulatory site of CmhR was identified in the promoter region of CmhR-regulated genes and this CmhR site was further confirmed by promoter mutational experiments.

## Data Summary

1. The sequence data of *S. pneumonaie* D39 which is used to construct all isolates in this study are available for download from http://www.ncbi.nlm.nih.gov/nuccore/NC_008533.1.

All supporting data, code and protocols have been provided within the article or through supplementary data files.

## Impact Statement

This study demonstrates methionine-mediated gene regulation in *Streptococcus pneumoniae* and identifies methionine transport and biosynthesis genes. *S. pneumoniae* is a human nasopharyngeal pathogen that is responsible for millions of deaths each year. Methionine is one of the important amino acids for pneumococci and some of the methionine-regulated genes have been shown to have a role in virulence in different bacteria including *S. pneumoniae*. In other bacteria, two to three transcriptional regulators have been shown to be involved in the regulation of sulphur-containing amino acids. The current study highlights the transcriptomic response of *S. pneumoniae* to methionine and identifies an important transcriptional regulator, CmhR, which acts as an activator for its regulon genes. The regulatory site of CmhR in the promoter regions of its regulon genes is predicted and confirmed through mutagenesis studies. CmhR (also called MtaR in other bacteria) has been demonstrated to have a role in virulence in many bacteria. Therefore, investigation of the involvement of CmhR in virulence in *S. pneumoniae* might be of interest.

## Introduction

*Streptococcus pneumoniae* colonizes the human nasopharynx during the first few months of life and is the causative agent of many human diseases including pneumonia, sepsis, meningitis, otitis media and conjunctivitis, resulting in over a million deaths each year worldwide (Gray *et al.*, 1982; Ispahani *et al.*, 2004; O’Brien *et al.*, 2009). Proper utilization of the available nutrients is a prerequisite for the successful colonization and survival of bacteria inside the human body in addition to the virulence factors it possesses (Phillips *et al.*, 1990; Titgemeyer & Hillen, 2002). Virulence gene screening and targeted examination of specific nutrient transporters have established the importance of nutrient acquisition for the pathogenesis of many microbial pathogens (Darwin, 2005; Lau *et al.*, 2001; Mei *et al.*, 1997). Amino acids are one of the most important groups of nutrients needed for proper bacterial growth.

Methionine is an amino acid that is scarcely present in physiological fluids but its importance cannot be underestimated. It is vital for protein synthesis and is an integral component of *S*-adenosyl methionine (SAM: the main biological methyl donor needed for the biosynthesis of phospholipids and nucleic acids) (Fontecave *et al.*, 2004; Shelver *et al.*, 2003). A number of genes/gene clusters are present in *S. pneumoniae* that can synthesize methionine from other sources in the absence of methionine. Csd and MetE are part of the methionine synthesis pathway and are involved in conversion of cystathionine to homocysteine, and homocysteine to methionine, respectively (Kanehisa *et al.*, 2014). Cystathionine and homocysteine can also be formed from homoserine, where *O*-acetyl-l-homoserine is converted to cystathionine by MetB. *O*-Acetyl-l-homoserine can be converted to homocysteine by MetB, SPD-1073 and SPD-1074 (*spd-1073* and *spd-1074* encode an *O*-acetylhomoserine aminocarboxypropyltransferase/cysteine synthase and a hypothetical protein, respectively) (Kanehisa *et al.*, 2014).

Methionine can be synthesized by other microbes as they may convert homoserine to homocysteine through addition of a sulphur group from either cysteine (involving MetABC), sulphide (involving MetA and CysD) or methionine using the SAM recycling pathway (MetK, Pfs and LuxS) (Kovaleva & Gelfand, 2007). MetE (methionine synthase) then methylates homocysteine in combination with MetF (methylenetetrahydrofolate reductase), and 5-methyltetrahydrofolate (FolD) provides it with the methyl group to form methionine (Kovaleva & Gelfand, 2007; Ravanel *et al.*, 1998). It has been shown that methionine biosynthetic genes are essential for full virulence in *Brucella melitensis* (Lestrate *et al.*, 2000), *Haemophilus parasuis* (Hill *et al.*, 2003) and *Salmonella enterica* (Ejim *et al.*, 2004). Moreover, mutation of the methionine transport regulator MtaR led to attenuated virulence in *Streptococcus agalactiae* (Shelver *et al.*, 2003), which suggests that methionine synthesis is indispensable for the existence of many bacteria during invasive infection.

In the current study, we elucidated the effect of methionine on the global gene expression of *S. pneumoniae* and demonstrated that the transcriptional regulator CmhR (SPD-0588) acts as a transcriptional activator for *fhs*, *folD*, *metB-csd*, *metEF*, *metQ* and *spd-0431*, involved in methionine uptake and utilization. The putative regulatory site of CmhR (5′-TATAGTTTSAAACTATA-3′, where S denotes G/C/A) in the promoter regions of its regulon genes is predicted and confirmed by promoter mutational experiments. This site is also found to be highly conserved in other pneumococcal strains and streptococci.

## Methods

### Bacterial strains, growth conditions and DNA isolation and modification.

Bacterial strains and plasmids used in this study are listed in Table 1. *S. pneumoniae* D39 wild-type was grown as described previously (Afzal *et al.*, 2014). For *β*-galactosidase assays, derivatives of *S. pneumoniae* D39 were grown in chemically defined medium (CDM) (Neves *et al.*, 2002), supplemented with different concentrations of methionine as indicated in the Results. For selection on antibiotics, the medium was supplemented with the following concentrations of antibiotics: spectinomycin at 150 µg ml^−1^ and tetracycline at 2.5 µg ml^−1^ for *S. pneumoniae*; and ampicillin at 100 µg ml^−1^ for *Escherichia coli*. All bacterial strains used in this study were stored in 10 % (v/v) glycerol at −80 °C. For PCR amplification, chromosomal DNA of *S. pneumoniae* D39 (Lanie *et al.*, 2007) was used as a template. Primers used in this study are based on the sequence of the D39 genome and are listed in Table S1, available in the online Supplementary Material.

**Table 1.** List of strains and plasmids used in this study

Strain/plasmid	Description	Source
*S. pneumoniae*		
D39	Serotype 2 strain. *cps 2*	Laboratory of P. Hermans
MA1100	D39 Δ*cmhR*; Spec^R^	This study
MA1101	D39 Δ*bgaA*:: P*spd-0150-lacZ*; Tet^R^	This study
MA1102	D39 Δ*bgaA*:: P*metQ-lacZ*; Tet^R^	This study
MA1103	D39 Δ*bgaA*:: P*spd-0431-lacZ*; Tet^R^	This study
MA1104	D39 Δ*bgaA*:: P*metE-lacZ*; Tet^R^	This study
MA1105	D39 Δ*bgaA*:: P*gshT-lacZ*; Tet^R^	This study
MA1106	D39 Δ*bgaA*:: P*spd-0618-lacZ*; Tet^R^	This study
MA1107	D39 Δ*bgaA*:: P*folD-lacZ*; Tet^R^	This study
MA1108	D39 Δ*bgaA*:: P*fhs-lacZ*; Tet^R^	This study
MA1109	D39 Δ*bgaA*:: P*tcyB-lacZ*; Tet^R^	This study
MA1110	D39 Δ*bgaA*:: P*metA-lacZ*; Tet^R^	This study
MA1111	D39 Δ*bgaA*:: P*metB-lacZ*; Tet^R^	This study
MA1112	MA1100 Δ*bgaA*:: P*metQ-lacZ*; Tet^R^	This study
MA1113	MA1100 Δ*bgaA*:: P*spd-0431-lacZ*; Tet^R^	This study
MA1114	MA1100 Δ*bgaA*:: P*metE-lacZ*; Tet^R^	This study
MA1115	MA1100 Δ*bgaA*:: P*folD-lacZ*; Tet^R^	This study
MA1116	MA1100 Δ*bgaA*:: P*fhs-lacZ*; Tet^R^	This study
MA1117	MA1100 Δ*bgaA*:: P*metB-lacZ*; Tet^R^	This study
MA1118	D39 Δ*bgaA*:: P*folD-M-lacZ*; Tet^R^	This study
MA1119	D39 Δ*bgaA*:: P*fhs-M-lacZ*; Tet^R^	This study
MA1120	D39 Δ*bgaA*:: P*metB-M-lacZ*; Tet^R^	This study
*E. coli*		
EC1000	Km^R^; MC1000 derivative carrying a single copy of the pWV1 *repA* gene in *glgB*	Laboratory collection
Plasmids		
pPP2	Amp^R^ Tet^R^; promoter-less *lacZ*. For replacement of *bgaA* with promoter *lacZ* fusion. Derivative of pPP1	Halfmann *et al.* (2007)
pMA1101	pPP2 P*spd-0150-lacZ*	This study
pMA1102	pPP2 P*metQ-lacZ*	This study
pMA1103	pPP2 P*spd-0431-lacZ*	This study
pMA1104	pPP2 P*metE-lacZ*	This study
pMA1105	pPP2 P*gshT-lacZ*	This study
pMA1106	pPP2 P*spd-0618-lacZ*	This study
pMA1107	pPP2 P*folD-lacZ*	This study
pMA1108	pPP2 P*fhs-lacZ*	This study
pMA1109	pPP2 P*tcyB-lacZ*	This study
pMA1110	pPP2 P*metA-lacZ*	This study
pMA1111	pPP2 P*metB-lacZ*	This study
pMA1112	pPP2 P*folD-M-lacZ*	This study
pMA1113	pPP2 P*fhs-M-lacZ*	This study
pMA1114	pPP2 P*metB-M-lacZ*	This study

### Construction of a *cmhR* mutant.

A *cmhR* (*spd-0588*) deletion mutant (MA1100) was made by allelic replacement with a spectinomycin-resistance cassette. Briefly, primers cmhR-1/cmhR-2 and cmhR-3/cmhR-4 were used to generate PCR fragments of the left and right flanking regions of *cmhR*. PCR products of left and right flanking regions of *cmhR* contain *Asc*I and *Not*I restriction enzyme sites, respectively. The spectinomycin-resistance marker, which is amplified by primers SpecR/SpecF from pORI38, also contains *Asc*I and *Not*I restriction enzyme sites on its ends. Then, by restriction and ligation, the left and right flanking regions of *cmhR* were fused to the spectinomycin-resistance gene. The resulting ligation products were transformed to *S. pneumoniae* D39 wild-type and selection of the mutant strains was done with the appropriate concentration of spectinomycin.

For transformation, cells were grown at 37 °C until an OD_600_ of ~0.1. Then, 0.2 % BSA and 1 mM CaCl_2_ were added to the cells. A 1 ml aliquot of the grown culture was transferred to a 1.5 ml tube and 100 ng µl^−1^ of CSP1 (Competence Stimulating Peptide 1) was added to the culture. Cells were incubated at 37 °C for 10–12 min. Then, the ligation mixture was added to the incubated cells and the cells were allowed to grow for 90–120 min at 37 °C. After growth, the culture was spun for 1 min at 7000 r.p.m. and most of the supernatant was discarded. The cell pellet was dissolved in the remaining medium (50–100 µl) and plated on blood agar plates with 1 % sheep blood. The *cmbR* mutant was further confirmed by PCR and DNA sequencing.

### Construction of promoter *lacZ*-fusions and *β*-galactosidase assays.

Chromosomal transcriptional *lacZ*-fusions to the *spd-0150*, *metQ* (*spd-0151*), *spd-0431*, *metE* (*spd-0510*), *gshT* (*spd-0540*), *spd-0618*, *folD* (*spd-0721*), *fhs* (*spd-1087*), *tcyB* (*spd-1290*), *metA* (*spd-1406*) and *metB* (*spd-1353*) promoters were constructed in the integration plasmid pPP2 (Halfmann *et al.*, 2007) with primer pairs mentioned in Table S1, resulting in pMA1101–1111, respectively. These constructs were further introduced into *S. pneumoniae* D39 wild-type, resulting in strains MA1101–11, respectively. pMA1102, pMA1103, pMA1104, pMA1107, pMA1108 and pMA1111 were also transformed into the D39 Δ*cmhR* strain resulting in strains MA1112–17, respectively. The following sub-clones of PfolD, Pfhs and PmetB with mutations in the *cmhR* site were made in pPP2 (Halfmann *et al.*, 2007) using the primer pairs mentioned in Table S1: P*folD*-M, P*fhs*-M and P*metB*-M, resulting in plasmids pMA1112–14, respectively. These constructs were introduced into the *S. pneumoniae* D39 wild-type, resulting in strains MA1118–20, respectively. All plasmid constructs were further checked for the presence of insert by PCR and DNA sequencing.

The *β*-galactosidase assays were performed as described before (Halfmann *et al.*, 2007; Israelsen *et al.*, 1995) using cells that were harvested in the mid-exponential growth phase and grown in CDM.

### Microarray analysis.

Microarray analysis was performed as described before (Afzal *et al.*, 2015a; Shafeeq *et al.*, 2015). For DNA microarray analysis of *S. pneumoniae* in the presence of methionine, the transcriptome of *S. pneumoniae* D39 wild-type, grown in replicates in CDM with 10 mM methionine, was compared to that grown in CDM with 0 mM methionine, and harvested at respective mid-exponential growth phases. For the identification of differentially expressed genes a Bayesian *P*-value <0.001 and a fold-change cut-off >1.5 was applied.

For RNA isolation the following procedure was performed: the pellet of the harvested cells was resuspended in 400 µl TE buffer (diethylpyrocarbonate) and the resuspended cells were added into RNA-free screw-cap tubes containing 0.5 g glass beads, 50 µl 10 % SDS, 500 µl phenol/chloroform: isoamylalcohol, macaloid layer (150–175 µl, not exact as it is highly viscous). To break the cells the screw-cap tubes were placed in a bead beater and two 1 min pulses were applied with 1 min interval on ice. The samples were centrifuged for 10 min at 10 000 r.p.m. (4 °C). Then, 500 µl chloroform/isoamylalcohol (24 : 1) was added to the tubes containing the upper phase of the centrifuged tubes and the samples were again centrifuged for 5 min at 10 000 r.p.m. (4 °C). A 500 µl aliquot of the upper phase was transferred to fresh tubes and total RNA was isolated using the High pure RNA isolation kit (Roche life science) according to the manufacturer’s instructions. RNA quality was assessed on a chip using an Agilent 2100 Bioanalyzer according to the manufacturer’s instructions and an RNA integrative number (RIN) value above 8 was considered good. All other procedures regarding the DNA microarray experiment and data analysis were performed as previously described (Afzal *et al.*, 2015b; Shafeeq *et al.*, 2011a, b). Microarray data have been submitted to GEO under accession number GSE88766.

### Reverse transcription (RT)-PCR and purification for quantitative RT-PCR.

For quantitative RT-PCR, *S. pneumoniae* D39 wild-type and D39 ∆*cmhR* were grown in CDM. RNA isolation was done as described above. First, cDNA synthesis was performed on RNA (Shafeeq *et al.*, 2011b; Yesilkaya *et al.*, 2008). cDNA (2 µl) was amplified in a 20 µl reaction volume that contained 3 pmol of each primer (Table S1) and the reactions were performed in triplicate (Shafeeq *et al.*, 2011b). The transcription level of specific genes was normalized to *gyrA* transcription, amplified in parallel with primers gyrA-F and gyrA-R. The results were interpreted using the comparative CT method (Schmittgen & Livak, 2008). Differences in expression of twofold or greater relative to the control were considered significant.

## Results

### Methionine-dependent gene regulation in *S. pneumoniae* D39

**Fig. 1.**
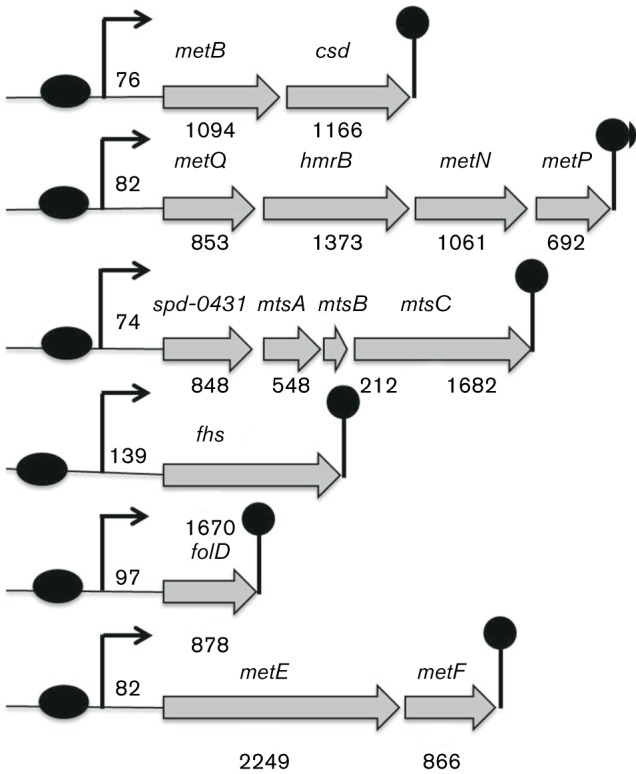
Organization of the CmhR-regulated genes in *S. pneumoniae* D39. Black ovals represent the putative CmhR binding sites, whereas the lollipop structures represent the putative transcriptional terminators. Numbers below genes represent the number of base pairs of genes, whereas the number between putative binding sites and start of genes shows the number of bases between the translation start sites and the putative CmhR binding sites. See text for further details.

The importance of methionine acquisition and synthesis for *S. pneumoniae* growth and virulence has been reported before (Basavanna *et al.*, 2013). In this study, we explored the impact of methionine on the transcriptome of *S. pneumoniae* D39. To do so, we performed transcriptome comparison of *S. pneumoniae* D39 wild-type grown in CDM with 0–10 mM methionine. The concentration of methionine in CDM is around 0.67 mM. A number of genes/gene clusters were differentially regulated under our tested conditions (Table 2). Putative methionine pathway genes were significantly upregulated in the absence of added methionine in CDM. These genes include *spd-0150* [coding for a predicted glutathione ATP binding cassette (ABC) transporter, substrate-binding protein], *metQ–hmrB–metNP* (a gene cluster putatively involved in methionine transport, whereas *hmrB* codes for a putative *N*-acyl-l-amino acid amidohydrolase), *spd-0431* (hypothetical protein), *metEF* (coding for 5-methyltetrahydropteroyltriglutamate-homocysteine methyltransferase and 5,10-methylenetetrahydrofolate reductase, respectively), *gshT* (coding for a predicted glutathione ABC transporter, substrate-binding protein), *spd-0616–18* (coding for a predicted polar amino acid ABC transporter), *folD* [coding for a methylenetetrahydrofolate dehydrogenase (NADP+)], *fhs* (coding for a formate-tetrahydrofolate ligase), *metA* (coding for a homoserine *O*-succinyltransferase), *metB–csd* (coding for a cystathionine gamma-synthase and cysteine desulfurase, respectively) and *tcyBC* (coding for a cysteine ABC transporter). The genetic organization of these putative methionine genes is shown in Fig. 1. There were some other genes (*spd-0360–63*, *spd-0372–74*, *spd-0385–91*, *spd-0447–49*, *spd-0616–18*, *spd-1073–75*, *spd-1098–99* and *spd–2037*) whose expression was also altered under our tested conditions and whose role in methionine biosynthesis and transport may be of interest. Some of the genes differentially regulated in our transcriptome comparison are putatively involved in carbohydrate transport and utilization (*spd-0424–28* and *spd-0559–62*), and these genes have been studied in our previous studies (Afzal *et al.*, 2014; Shafeeq *et al.*, 2012). The *spd-0424–28* genes putatively encode a cellobiose/lactose-specific phosphotransferase system, and transcriptional regulator RokA acts as a transcriptional repressor of this operon (Shafeeq *et al.*, 2012). The *spd-0559–62* genes putatively code for a lactose/galactose-specific phosphotransferase system and the regulatory mechanism of this gene cluster is not yet known. A gene, *spd-0588*, coding for a transcriptional regulator is also upregulated in our methionine microarray. Our bioinformatics analysis shows that this gene shares high homology with *mtaR* present in other bacteria and because of its putative involvement in cysteine and methionine metabolism we call it *cmhR*. The upregulation of *cmhR* under our tested conditions might be an indication of its involvement in the regulation of the methionine genes.

**Table 2.** Summary of transcriptome comparison of *S. pneumoniae* D39 wild-type grown in CDM with 0–10 mM methionine PTS, phosphotransferase system.

D39 tag*	Function†	Ratio‡
**Upregulated genes**		
*spd-0152*	Peptidase, M20/M25/M40 family protein	2.9
*spd-0153*	ABC transporter, ATP-binding protein	2.3
*spd-0154*	ABC transporter, permease protein, putative	1.9
*spd-0360*	PTS system, mannitol-specific IIBC components	3.4
*spd-0361*	Transcriptional regulator, putative	3.0
*spd-0362*	PTS system, mannitol-specific enzyme IIA	2.8
*spd-0363*	Mannitol-1-phosphate 5-dehydrogenase	3.2
*spd-0372*	Sodium:alanine symporter family protein	2.2
*spd-0373*	Hypothetical protein	4.8
*spd-0374*	Exfoliative toxin, putative	2.1
*spd-0424*	PTS system, cellobiose-specific IIC component	2.6
*spd-0426*	PTS system, lactose-specific IIA component	2.0
*spd-0427*	6-Phospho-*β*-galactosidase	2.5
*spd-0428*	PTS system, lactose-specific IIBC components	2.5
*spd-0429*	Potassium uptake protein, Trk family protein	2.4
*spd-0430*	Potassium uptake protein, Trk family protein	2.0
*spd-0431*	Hypothetical protein	1.8
*spd-0432*	Hypothetical protein	1.8
*spd-0434*	ABC transporter, ATP-binding protein	1.6
*spd-0510*	5-Methyltetrahydropteroyltriglutamate-homocysteine *S*-methyltransferase, MetE	5.0
*spd-0511*	5,10-Methylenetetrahydrofolate reductase, MetF	7.7
*spd-0512*	Polyribonucleotide nucleotidyltransferase	1.8
*spd-0513*	Serine *O*-acetyltransferase	1.6
*spd-0514*	Acetyltransferase, GNAT family protein	1.8
*spd-0515*	Cysteinyl-tRNA synthetase	1.5
*spd-0540*	Amino acid ABC transporter, amino acid-binding protein, putative	2.4
*spd-0559*	PTS system IIA component, putative	2.0
*spd-0560*	PTS system, IIB component, putative	1.5
*spd-0561*	PTS system, IIC component, putative	2.2
*spd-0562*	*β*-Galactosidase precursor, putative	2.5
*spd-0588*	Transcriptional regulator, putative, CmhR	1.5
*spd-0610*	Hypothetical protein	2.5
*spd-0611*	Hypothetical protein	2.4
*spd-0612*	Lipoprotein, putative	2.2
*spd-0613*	Hypothetical protein	2.6
*spd-0614*	ABC transporter, ATP-binding protein	4.2
*spd-0616*	Amino acid ABC transporter, ATP-binding protein	1.7
*spd-0617*	Amino acid ABC transporter, permease protein	2.3
*spd-0618*	Amino acid ABC transporter, permease protein	1.7
*spd-0818*	Transcriptional regulator, LysR family protein	2.1
*spd-1073*	*O*-Acetylhomoserine aminocarboxypropyltransferase/cysteine synthase	2.3
*spd-1074*	Hypothetical protein	2.3
*spd-1075*	Transporter, FNT family protein, putative	2.0
*spd-1087*	Formate-tetrahydrofolate ligase, Fhs	1.7
*spd-1351*	Snf2 family protein	1.5
*spd-1352*	Aminotransferase, class II, Csd	3.5
*spd-1353*	Cys/Met metabolism PLP-dependent enzyme, putative, MetB	1.5
*spd-1355*	Hypothetical protein	2.4
*spd-1899*	Glutamine amidotransferase, class 1	1.6
**Downregulated genes**		
*spd-0147*	CAAX amino terminal protease family protein	−2.0
*spd-0278*	Hypothetical protein	−2.2
*spd-0279*	PTS system, IIB component	−2.6
*spd-0281*	PTS system, IIA component	−4.3
*spd-0282*	Hypothetical protein	−3.8
*spd-0283*	PTS system, IIC component	−3.4
*spd-0385*	3-Oxoacyl-[acyl-carrier-protein] synthase II	−2.1
*spd-0386*	Acetyl-CoA carboxylase, biotin carboxyl carrier protein	−2.5
*spd-0387*	*β*-Hydroxyacyl-(acyl-carrier-protein) dehydratase FabZ	−2.2
*spd-0388*	Acetyl-CoA carboxylase, biotin carboxylase	−2.1
*spd-0389*	Acetyl-CoA carboxylase, carboxyl transferase, beta subunit	−4.1
*spd-0390*	Acetyl-CoA carboxylase, carboxyl transferase, alpha subunit	−2.3
*spd-0391*	Hypothetical protein	−2.3
*spd-0447*	Transcriptional regulator, GlnR	−2.1
*spd-0448*	Glutamine synthetase, GlnA	−2.5
*spd-0449*	Hypothetical protein	−2.1
*spd-0636*	Pyruvate oxidase, SpxB	−3.6
*spd-1041*	Glutaredoxin-like protein, NrdH	−3.6
*spd-1042*	Ribonucleoside-diphosphate reductase, NrdE	−3.0
*spd-1098*	Amino acid ABC transporter, GlnP	−2.1
*spd-1099*	Amino acid ABC transporter, GlnQ	−2.0
*spd-1461*	Manganese ABC transporter, ATP-binding protein, PsaB	−2.7
*spd-1463*	ABC transporter, substrate binding lipoprotein	−2.3
*spd-1464*	Thiol peroxidase, PsaD	−2.2
*spd-2037*	Cysteine synthase A, CysK	−1.8

*Gene numbers refer to D39 locus tags.

†D39 annotation/TIGR4 annotation (Lanie *et al.*, 2007).

‡Ratio represents the fold increase/decrease in the expression of genes in CDM with 0–10 mM methionine. Errors in the ratios never exceeded 10 % of the given values.

### Confirmation of methionine-dependent expression of *fhs*, *folD*, *gshT*, *metA*, *metB*, *metEF*, *metQ*, *tcyB*, *spd-0150*, *spd-0431* and *spd-0618*

**Fig. 2.**
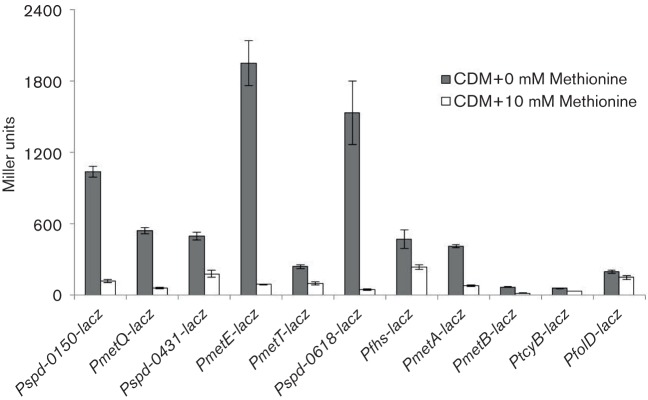
Expression levels (in Miller units) of P*spd-0150-lacZ*, P*metQ-lacZ*, P*spd-0431-lacZ*, P*metE-lacZ*, P*gshT-lacZ*, P*spd-0618-lacZ*, P*fhs-lacZ*, P*metA-lacZ*, P*metB-lacZ*, P*tcyB-lacZ* and P*folD-lacZ* in *S. pneumoniae* D39 wild-type grown in CDM with 0 and 10 mM methionine. Standard deviations of three independent experiments are indicated by bars.

To confirm our microarray results and study the expression of *fhs*, *folD*, *gshT*, *metA*, *metB–csd*, *metEF*, *metQ*, *tcyB*, *spd-0150*, *spd-0431* and *spd-0618* in the presence and absence of methionine in CDM, we constructed promoter lacZ-fusions of these putative methionine genes and transformed these promoter *lacZ*-fusions into *S. pneumoniae* D39 wild-type. *β*-Galactosidase assays were performed on cells grown in CDM with 0 and 10 mM methionine (Fig. 2). Our *β*-galactosidase assay results showed that expression of *fhs*, *folD*, *gshT*, *metA*, *metB-csd*, *metEF*, *metQ*, *tcyB*, *spd-0150*, *spd-0431* and *spd-0618* promoters increased significantly in the absence of added methionine in CDM. These data further confirm our microarray data mentioned above.

### Prediction of the CmhR regulatory site in CmhR-regulated genes

**Fig. 3.**
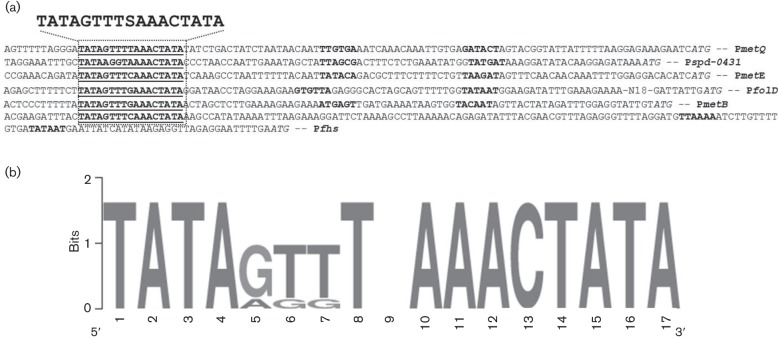
Identification of the CmhR regulatory site. (a) Weight matrix of the identified CmhR regulatory site in the promoter region of *metQ*, *spd-0431*, *metE*, *folD*, *metB* and *fhs*. (b) Position of the CmhR regulatory site in the promoter region of *metQ*, *spd-0431*, *metE*, *folD*, *metB* and *fhs*. Core promoter sequences are in bold, translational start sites are in italic and putative CmhR regulatory sites are bold-underlined.

A number of genes/gene clusters were differentially regulated under our tested conditions. One important gene that was significantly upregulated in our microarray results was *cmhR* (*spd-0588*). This gene codes for a transcriptional regulator CmhR, which putatively belongs to the LysR family of proteins (Shelver *et al.*, 2003). CmhR is a homologue of MtaR of other bacteria and our bioinformatics analysis shows that the MtaR binding site is also present in the genes of the CmhR regulon in *S. pneumoniae*. Using Genome2D software (Baerends *et al.*, 2004) and a MEME motif sampler search (Bailey & Elkan, 1994), a 17 bp palindromic sequence was found in the promoter regions of several genes in *S. pneumoniae* D39. These genes were identified in our methionine microarray suggesting their role in methionine transport and metabolism. The CmhR regulatory site present in the promoter regions of methionine genes is shown in Fig. 3(a). A weight matrix of these putative CmhR regulatory sites (5′-TATAGTTTSAAACTATA-3′) was constructed using these DNA regions (Fig. 3b). This DNA sequence may serve as the CmhR regulatory site in *S. pneumoniae*. Promoter regions of these genes were also examined in other streptococcal species (*Streptococcus mitis*, *Streptococcus gordonii*, *Streptococcus mutans*, *Streptococcus thermophiles*, *Streptococcus uberis*, *Streptococcus agalactiae*, *Streptococcus gallolyticus*, *Streptococcus sanguinis* and *Streptococcus suis*) to check if the CmhR regulatory site is also conserved in those streptococci. From this study, we conclude that the CmhR regulatory sequence is highly conserved in these streptococci as well (Fig. S1).

### CmhR acts as transcriptional activator of *metQ*, *spd-0431*, *metEF*, *folD*, *fhs* and *metB-csd* in *S. pneumoniae* D39

**Fig. 4.**
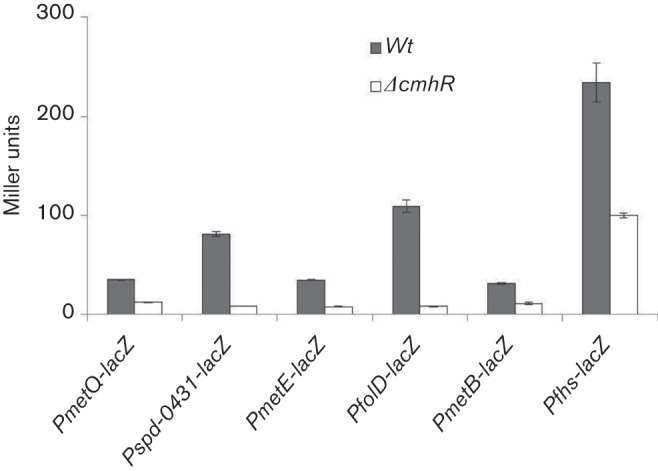
Expression levels (in Miller units) of P*metQ-lacZ*, P*spd-0431-lacZ*, P*metE-lacZ*, P*folD-lacZ*, P*metB-lacZ* and P*fhs-lacZ* in *S. pneumoniae* D39 wild-type and D39 ∆*cmhR* grown in CDM. Standard deviations of three independent experiments are indicated as bars.

The genes that are proposed to be the part of the CmhR regulon are *metQ*, *spd-0431*, *metEF*, *folD*, *fhs* and *metB-csd*. To investigate the role of CmhR in the regulation of the proposed CmhR regulon, we made a *cmhR* deletion mutant and transformed the *lacZ-*fusions of the promoter regions of these genes in to D39 ∆*cmhR* and performed *β*-galactosidase assays in CDM (Fig. 4). The results of the *β*-galactosidase assays showed that the activity of all these promoters decreased significantly in D39 *∆cmhR* compared to the D39 wild-type, suggesting a role for CmhR as a transcriptional activator of these genes.

**Fig. 5.**
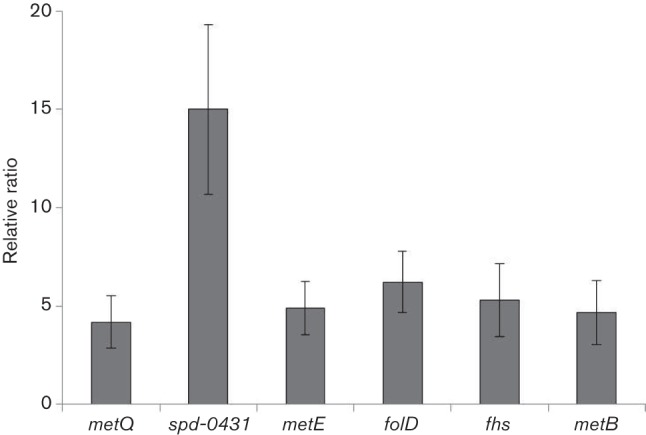
The relative increase in the expression of *metQ*, *spd-0431*, *metE*, *folD*, *fhs* and *metB* in *S. pneumoniae* D39 wild-type compared to D39 Δ*cmhR* grown in CDM. Expression of *metQ*, *spd-0431*, *metE*, *folD*, *fhs* and *metB* was normalized with the housekeeping gene *gyrA*. Results represent the mean and standard deviation of three independent replicates.

To further confirm our *β*-galactosidase assay results mentioned above, we performed quantitative RT-PCR on CmhR-regulated genes (*metQ*, *spd-0431*, *metEF*, *folD*, *fhs* and *metB*). Our quantitative RT-PCR results also demonstrated that the expression of these genes increased significantly in the D39 wild-type compared to D39 ∆*cmhR* (Fig. 5). These results further confirm that CmhR acts as a transcriptional activator of *metQ*, *spd-0431*, *metEF*, *folD*, *fhs* and *metB-csd*.

### Verification of a CmhR regulatory site in CmhR-regulated genes

**Fig. 6.**
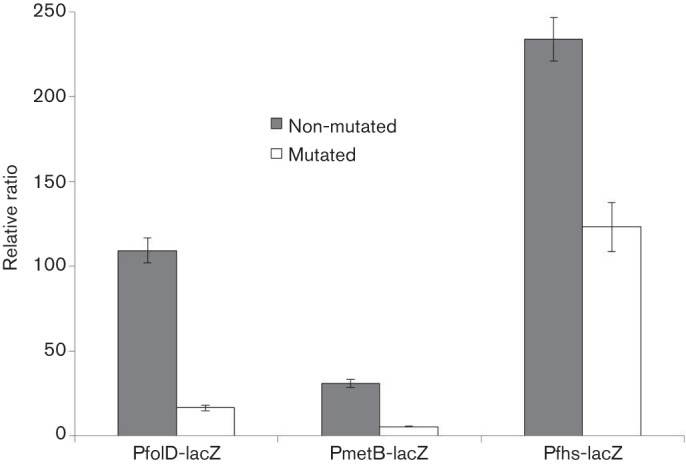
Expression levels (in Miller units) of mutated and non-mutated CmbR regulatory site in P*folD-lacZ*, P*metB-lacZ* and P*fhs-lacZ* in *S. pneumoniae* D39 wild-type grown in CDM. Standard deviations of three independent experiments are indicated as bars.

To verify the CmhR regulatory site present in the promoter regions of the CmhR-regulated genes (*metQ*, *spd-0431*, *metEF*, *folD*, *fhs* and *metB-csd*), we made transcriptional *lacZ*-fusions of P*folD*, P*fhs* and P*metB*, where conserved bases in the putative *cmhR* regulatory sites were mutated (shown in bold-underlined) in P*folD* (5′-T**ATA**GTTTGAAAC**TAT**A-3′ to 5′-T**CGC**GTTTGAAAC**GCG**A-3′), P*fhs* (5′-T**ATA**GTTTCAAAC**TAT**A-3′ to 5′-T**CGC**GTTTCAAAC**GCG**A-3′) and P*metB* (5′-T**ATA**GTTTGAAAC**TAT**A-3′ to 5′-T**CGC**GTTTGAAAC**GCG**A-3′), and *β*-galactosidase assays were performed with cells grown in CDM. The expression of these promoters with mutated conserved bases in CmhR regulatory sites decreased significantly. These results confirm that the predicted CmhR sites present in the promoter regions of these genes are active and intact in *S. pneumoniae* D39 (Fig. 6).

## Discussion

Bacterial pathogens need to acquire the essential nutrients from their surroundings inside the human host in order to survive and cause infections (Eisenreich *et al.*, 2010; Kilian *et al.*, 1979). In this study, we have explored the impact of methionine on the gene expression of *S. pneumoniae* D39 and found that a number of genes/operons respond to methionine. The results presented in this study will significantly enhance our understanding of the role of methionine on the gene expression of *S. pneumoniae*.

Several regulatory systems involved in the regulation of methionine biosynthesis have been reported in bacteria. In most Gram-positive bacteria, RNA structures acting on the level of premature termination of transcription control methionine biosynthesis: S-boxes present upstream of methionine biosynthesis genes in the *Bacillales* and *Clostridia* (Epshtein *et al.*, 2003; McDaniel *et al.*, 2003; Winkler *et al.*, 2003), and methionine-specific T-boxes in the *Lactobacillales* (Grundy & Henkin, 2003; Rodionov *et al.*, 2004). Conversely, neither S-boxes nor methionine-specific T-boxes were found in *Streptococcaceae* for all methionine biosynthesis genes, with *metK* being the only exception that is regulated by the SMK-riboswitch (Fuchs *et al.*, 2006). Therefore, it is clear that methionine biosynthesis in the *Streptococcacae* is controlled by a different mechanism that evolved after the split of this group from other *Firmicutes*.

Three transcriptional factors have been found to play a role in regulation of methionine metabolism in streptococci: MtaR in *S. agalactiae*, its orthologue MetR in *S. mutans* and CmbR (earlier known as FhuR) in *Lactococcus lactis* (Fernández *et al.*, 2002). Methionine uptake decreased fivefold in the *mtaR* mutant in comparison to the wild-type in *S. agalactiae*, suggesting its role as a transcriptional activator of the methionine transport genes (Shelver *et al.*, 2003). By contrast, MetJ and MetR regulate the expression of methionine biosynthetic genes in *E. coli* and *Salmonella enterica* serovar Typhimurium (Weissbach & Brot, 1991). The *E. coli met* genes (except for *metH*) are negatively regulated by MetJ, a transcriptional repressor, with SAM serving as a co-repressor (Saint-Girons *et al.*, 1988,). These genes are also positively regulated by a LysR-type transcriptional regulator MetR, with homocysteine as a co-effector (Cai *et al.*, 1989; Cowan *et al.*, 1993; Mares *et al.*, 1992). CmbR in *L. lactis* has been demonstrated to activate most genes involved in the methionine and cysteine biosynthesis pathway in the absence of cysteine (Fernández *et al.*, 2002; Sperandio *et al.*, 2005). MetR has also been shown to regulate a number of methionine biosynthesis genes (Sperandio *et al.*, 2007) by binding to the DNA motif suggested by Rodionov *et al.* (2004). The regulatory proteins mentioned above belong to the LysR family of transcriptional factors, which is the most abundant family of transcriptional regulators in bacteria (Maddocks & Oyston, 2008). These transcriptional regulators control diverse biological pathways such as central metabolism, cell division, quorum sensing, virulence, motility, nitrogen fixation, oxidative stress responses, toxin production, attachment and secretion. These transcriptional regulators act as either transcriptional activators or repressors, and often are transcribed divergently with one of the regulated genes (Schell, 1993). LysR-family regulators consist of two characteristic domains, an N-terminal helix–turn–helix DNA binding domain (PF00126) and a C-terminal substrate-binding domain (PF03466). There appear to be two transcriptional regulators in *S. pneumoniae* that control the expression of the cysteine and methionine genes. CmhR in *S. pneumoniae* belongs to the LysR family of transcriptional factors and has a helix–turn–helix domain and a substrate-binding domain of LysR-type transcriptional regulators. The second is CmbR and study of its regulatory mechanism might be of interest (*L. lactis* has also two: CmbR and CmhR). Our results showed that CmhR acts as a transcriptional activator for a number of genes involved in methionine uptake and biosynthesis and deletion of CmhR led to downregulation of the CmhR genes. The deletion of CmhR also hampers growth of *S. pneumoniae* (data not shown), which might be an indication of the importance of this protein in the lifestyle of pneumococci.

In this study, we have demonstrated that the CmhR regulon consists of *metQ*, *spd-0431*, *metEF*, *fhs*, *metB-csd* and *folD* in *S. pneumoniae* D39. There are two bacterial methionine transport systems: the methionine ABC uptake transporter (MUT) family (Hullo *et al.*, 2004; Merlin *et al.*, 2002) and a secondary transporter BcaP (den Hengst *et al.*, 2006). The MUT system is encoded by the *metD* locus in *E. coli* and consists of the MetQ substrate binding protein (SBP), MetL trans-membrane permease and the MetN cytoplasmic ATP-hydrolysing protein (ATPase) (Merlin *et al.*, 2002). In *S. pneumoniae* D39, the *spd-0150–54* locus encodes a methionine uptake ABC transporter and deletion of the gene encoding the lipoprotein MetQ resulted in a strain that had reduced growth in methionine-restricted media and no detectable uptake of radioactive methionine (Basavanna *et al.*, 2013). Moreover, deletion of another important locus encoding MetEF (which is also part of the CmhR regulon) increased the growth defect of the *metQ* deletion strain in methionine-restricted media and in blood plasma, strengthening a role for the products of these genes in methionine synthesis (Basavanna *et al.*, 2013). Micro-organisms can synthesize methionine by converting homoserine to homocysteine through addition of a sulphur group from either cysteine (requiring MetABC), sulphide (requiring MetA and CysD) or methionine using the SAM recycling pathway (MetK, Pfs and LuxS) (Kovaleva & Gelfand, 2007). Homocysteine is then methylated by methionine synthase (MetE) in conjunction with a methylenetetrahydrofolate reductase (MetF), with the methyl group supplied by 5-methyltetrahydrofolate, to form methionine (Kovaleva & Gelfand, 2007). Existing data show that methionine biosynthetic genes are required for the full virulence of *B. melitensis* (Lestrate *et al.*, 2000), *H. parasuis* (Hill *et al.*, 2003) and *Salmonella enterica* (Ejim *et al.*, 2004), and that mutation of the *S. agalactiae* methionine regulator MtaR attenuates virulence (Shelver *et al.*, 2003), suggesting methionine synthesis is essential for survival of many bacteria during invasive infection. These observations also suggest that CmhR might have a role in pneumococcal pathogenesis and further studies may shed more light on this. Our microarray results show that the genes discussed above are part of methionine biosynthesis and transport genes, and are differentially regulated in our methionine microarray. Therefore, further investigations of the CmhR regulon may provide valuable information regarding virulence mechanisms in *S. pneumoniae*, which could be very useful for devising strategies to combat pneumococcal infections.

## Supplementary Data

538 Supplementary File 1
