# Supplementary material for: Methionine-mediated gene expression and characterization of the CmhR regulon in Streptococcus pneumoniae
Source: Microb Genom. 2016 Oct 21;2(10):e000091. doi: 10.1099/mgen.0.000091 (PMC5359408; doi:10.1099/mgen.0.000091)
Supplement: Supplementary File 1 [file mgen-02-91-s001.pdf]

Supplementary material

Methionine-mediated gene expression and characterization of the CmhR regulon in *Streptococcus pneumoniae*

Muhammad Afzal, Sulman Shafeeq and Oscar P. Kuipers

Table S1: List of primers used in this study.

Name	Nucleotide Sequence (5’→3’)*	Restrictio
spd-0150-F	CATGGAATTCGCTAGACTTCTTAGCCGTC	*EcoRI*
spd-0150-R	CATGGGATCCGGCAGCAAGAGATGAGTAT	*BamHI*
metQ-F	CATGGAATTCCGGAGACACTTATCTACCGG	*EcoRI*
metQ-R	CATGGGATCCAACCTGCGACTGTAGCAAGG	*BamHI*
spd-0431-F	CATGGAATTCGACAATATGATTGAGAACAGC	*EcoRI*
spd-0431-R	CATGGGATCCCCAAAGTCTGATTGTAATACC	*BamHI*
metE-F	CATGGAATTCGCAGTCGTCCGACTTGAAC	*EcoRI*
metE-R	CATGGGATCCGTTGACATGATGTGTCCTCC	*BamHI*
gshT-F	CATGGAATTCGGATCAACAGGCTTATCAG	*EcoRI*
gshT-R	CATGGGATCCGGTAGGATAGCTAGTAATAGGC	*BamHI*
spd-0618-F	CATGGAATTCCTACATGACCTTCTGCATTGC	*EcoRI*
spd-0618-R	CATGGGATCCGTTCAACAATGGACCAATCC	*BamHI*
folD-F	CATGGAATTCGGATGACTATGGTTATCG	*EcoRI*
folD-R	CATGGGATCCCCATCAATAATCTGTGTCATATC	*BamHI*
fhs-F	CATGGAATTCCCAAGTGACCTGTAGAATGG	*EcoRI*
fhs-R	CATGGGATCCCAACTCAATACTCTGTGCGA	*BamHI*
tcyB-F	CATGGAATTCGTTAAGCAAATCGGTTATCG	*EcoRI*
tcyB-R	CATGGGATCCCCAATCTGATGCTAAAATCG	*BamHI*
metA-F	CATGGAATTCCGATACCTTGACTATGCACG	*EcoRI*
metA-R	CATGGGATCCGCACGTTGATCATCCATGAC	*BamHI*
metB-F	CCATGGAATTCGGAGCAAATGATAGAGTGAAGG	*EcoRI*
metB-R	CATGGGATCCCCTTGCTCATACAATACCTCC	*BamHI*
metQ-F	CATGGAATTCAGTTTTTAGGGATCGCGTTTTAAACGCGA	*EcoRI*
spd-0431-M-F	CATGGAATTCGGAAATTTGCTCGCAGGTAAAACGCGA	*EcoRI*
folD-M-F	CATGGAATTCGTTCAACCAGAGCTTTTTCTTCGCGTTTGAAACGCGA	*EcoRI*
fhs-M-F	CATGGAATTCGGTAAAAACGAAGATTTACTCGCGTTTCAAACGCGA	*EcoRI*
metB-M-F	CATGGAATTCGCAGGAAAAACTCCCTTTTTATCGCGTTTGAAACGCGA	*EcoRI*
CmhR-1	GCCAAGACCAATCCTATGATACC	-
CmhR-2	GCTATGGCGCGCCCCATACTTGCCTTAGAATCGC	*AscI*
CmhR-3	GCTAAGCGGCCGCCGCTTTATAGACTATCTCCTAG	*NotI*
CmhR-4	CCTACTATTCTCATTACCGGAGC	-
**RT-PCR Primers**		
metQ-1	GCTACAGTCGCAGGTTTGGC	-

metQ-2	CTTCGCCATCAGCAGTTGC	-
spd-0431-1	GGTCTGGTTGATGGTGCGG	-
spd-0431-2	CCAGTAATCCACCGTCTG	-
metE-1	GGCATCACTGAAATCCC	-
metE-2	GGTAACCACGTCCCAAAGCG	-
fhs-1	CAGATATTGAAATCGCACAG	-
fhs-2	GCCTTGTACTTTCCGTAC	-
folD-1	GAGAAGTGATATGACACAG	-
FolD-2	CGTAGACTTGGCTGGCTGG	-
metB-1	GTCAGATGAAGCGACAGG	-
metB-2	GCCAAGACTTCCTCAGCC	-

* Restriction enzyme sites are underlined.

TAGGGA**TATAGCTTTAAACTATA**TATCTGACTATCTAATAACAATTTGTGAAATCAAACAAATTGTGAGATACTGATACAGTATTATTTTTAAGGAGAAAGAATCATG—P*metQ* SM

TTTGC**TATAAGGTAAAACTATA**CCCTAACCAATTGAAATAGCTATTAGCACCTTTCTCTGAAATATGGTATGATAAAGGATATACAAGGAGATAAAATG—P *smi_1812* SM

AGAGA**TATAGTTTCAAACTATA**TCAAAGCCTAATTTTTTACAATTATACAGATGATTCCTTTTCTGTTAAGATAGTTTCAACAACAAATTTTGGAGGACACATCATG—P*metE* SM

TTTCT**TATAGTTTGAAACTATA**AGGTTGCCTAAGAAAGAAGT—P*folD* SM

AAGAG**TATAGTTTCAAACTATA**AATCCATATAAAAATTAAGAAAGAAGGCTATAATCCTTTAAAAACGCAAATTTTACGAACGTTAATAGAGTTCCTTTCTATTAAAAATCTGTTTTTTGTGTTATAATGA
ATTATCATATAAGAGGTTAGAGGAGTTTTGAATG—P*fhs* SM

TAGGT**TAAAGTTCAGGACTATA**AGAGGGAGAATACATG—P*spd-0431* SG

TAAGT**TATAGTTTTTGATTATA**CCAAACAATAATTTCAATCAATGTCAGTTGTAAAATAGGGTTTTCTAATGATCAAAAGAATCGGGATATAGTTTTTGGCTATATCAAAATTAGGGAAATTCCAATTATA
CAGAGGGTGGATTTTCTGATAAGATAAAATCAAGAAAAATTTTGGAGGACACATATG—P*metE* SG

TCTTT**TTTTGTTATAATCTATA**TACTAAATGGATGAAGGAGAATTTGATG—P*fhs* SG

TTTTA**TATAGTTTCAGACTATA**TCTAGCTCTTGAAAAGAACAAAGCGCAGAACTTTCGCAAGATGTTACAATAGAATAAGATTTACGAGATTGTGAGGAATAAGAAATG—P*metB* SG

ACAGT**TATAGGTAATAACTATA**GCTGTTTCTACTCTTTTACAGTCTACAAAAAACAATAATCTTGTGATAAGATAAATAAGAAGTAAATAAGAAAGAAGGGCAAAATTATTAAAAATG—P*smu.1936c* SN

TTTGT**TATAGATGAAAACTATA**ACAAATACTACATATATAGCTTAAAACTATACATATACATAAAAACACAAAAACCCTTGAAACATTGATTTAATAAGGATTATCTAGTATTATTTTAATTACAATAAAA
AAAGAAGTTATAGTTATAAGATGAATACAGTTTTTATCTTAAGGCTAAGAGGAGGACGAAATG—P*metE* SN

CTGAT**TATAATATTTAAATATA**GAAAAAAAGGTGTAGCAGCGTGTTATCTGCTCGCCTTTTTGGTATAATAAAAGCAGTATTTTTTCAGGAGATGAGAATG—P*folD* SN

GGAAA**CATAGCCAAAGGCTATA**ACTAGCTCAAGAATTTGTCATCTTTGAAATATCTATAAAAAGTGGTAAATTAAACATCTCTAGTAGAAAAAGGTGAGATACTTATG—P*str0304* ST

CCCTA**TATAGTTATTAGCTATA**CAAATATCTAATTAAAAATAAGACAACGATACCAGAAATCCTTTATTTTATAGGATATAGTTTAAAACTATATATATTGAAAACTGATAACAATTATACAGATATTTAT
TTCTTTG—P*metE* ST

GAGGT**TATAGTAATAAACTATA**TCTAACTATCAAGAATTCGTATGATGAACTTTCCATTCTTTTCAGAAAATAGTGATAAAATAGAGGGCATAGATATGGAGAATATCTATTTTATCATGTAGATAGGGTG
CAATATG—P*metB* ST

GACGT**TATAGTTTTAGACTATA**GATTTCCCCCTTCGATTCCTTATTTAGCTTGCCTTCTCTTTATGTTAGAATAAAGTTGTATCAACATTTTAATGAAAAGGGGAGTCTTAATG—P*SUB0314* SU

TTTAT**TAAAGATATACACTATA**TAGAAAGTTAGGTAATCTAAAAACGAACAATATAAAAAAAATAAATCTAAAAGAATAAGAAATTAAATTAAATATGCTATAATGGATTCAAGTTAATAATAAGGAGTTT
AGCATG—P*fhs* SU

GTTTA**TATAGTTTAAAACTATA**TAAATCGCTAAAAATATAAAATATTAAAAGCTATTGATATTATAGTTTTCATGGACTAAAATAATTCATAAAACTAAAAGGGTTAGTTATTAGCTATAACTTGATGGAG
GAAATTATG—P*metE* SA

TTTGT**TATAGTTAAAAACTATA**ATAAACTCTAAATGTATAGCTAAAAACTATATCAGGCCATAAAAAGATAGAATGATGAAAGGTATTGAAAAAAGGCTTGTTATCCTTTACAATCTTAATTATAAAAAAT
TTAGTAGGTATAGTTTAAAACTAATGCTGGTATAGGAGGTAACTATG—P*metE* SL

TTTAG**TTATAGTTTTTACTTAT**AGCTGATTATCAAGAAATAGAATAACGAAAAAGTCATGCATTTCAACGAATAAATGATAGAATAGTTTATAAATTTTAATAGACCAGTATAGCTGGTAGTGATATGTTT
TGAGGGGGAGACACCATG—P*metB* SL

AAGGT**CATAATTTCTACCTATA**GCAAACAATAATTTCAGTCAATGGTGCTTCTAAAATGGGGGTTTTGGACTGGTTGGCGAGATAGGTTATAGTTTAGACTATATCAAACTCTGGGAAATTCCAATTATA
CAGAGGGGGGATTTTCTGATAAGATAAAATCAAGAAAAATTTTGGAGGACGTTTATG—P*metE* SS

AACAT**TATAGTTTCAAACTATA**GCTAGCTCTTGAAAAGAAAAAAGAAAAATG—P*metB* SS

AGTTT**CATAGATAAAATCTATA**CCACGCTTAAGAAAAAACTATACTGGAGCCAGCATAAAAAAGTCAAGCTTGATAAACAGGCTTTTTTTCAAGCTTTTATATAGAAGAAATATCAAAAAAGTAGCTCTCT
TTCAATCTGTTATAGTCAAAAGTTATACCTCCCTCTAATTTTTTACAATTATACAAGAGTCGTTTTTTCTGTTAGGATAGGGGCATAGAAATATTTTGGAGGAAAAACCATG—P*metE* SI

SM= *S. mitis*, SG= S. *gordonii*, SN= *S. mutans*, ST= *S. thermophilus*, SU= *S. uberis*, SA= S. *agalactiae*, SL= *S. gallolyticus*, SS= *S. sanguis* and SI= *S. suis*.

Fig. S1: Promoter sequences of putative CmhR regulated genes in different Streptococci showing conservation of CmhR regulatory site.
